# Quantification of HER Expression and Dimerization in Patients’ Tumor Samples Using Time-Resolved Förster Resonance Energy Transfer

**DOI:** 10.1371/journal.pone.0037065

**Published:** 2012-07-19

**Authors:** Alexandre Ho-Pun-Cheung, Hervé Bazin, Nadège Gaborit, Christel Larbouret, Patrick Garnero, Eric Assenat, Florence Castan, Caroline Bascoul-Mollevi, Jeanne Ramos, Marc Ychou, André Pèlegrin, Gérard Mathis, Evelyne Lopez-Crapez

**Affiliations:** 1 Translational Research Unit, CRLC Val d’Aurelle Paul Lamarque, Montpellier, France; 2 Research Department, Cisbio Bioassays, Codolet, France; 3 Institut de Recherche en Cancérologie de Montpellier (IRCM), INSERM, U896, Université Montpellier1, CRLC Val d’Aurelle Paul Lamarque, Montpellier, France; 4 Department of Medical and Digestive Oncology, CRLC Val d’Aurelle Paul Lamarque, Montpellier, France; 5 Department of Biostatistics, CRLC Val d’Aurelle Paul Lamarque, Montpellier, France; 6 Department of Pathology, Center Hospital University, Montpellier, France; Health Canada, Canada

## Abstract

Following the development of targeted therapies against EGFR and HER2, two members of the human epidermal receptor (HER) family of receptor tyrosine kinases, much interest has been focused on their expression in tumors. However, knowing the expression levels of individual receptors may not be sufficient to predict drug response. Here, we describe the development of antibody-based time-resolved Förster resonance energy transfer (TR-FRET) assays for the comprehensive analysis not only of EGFR and HER2 expression in tumor cryosections, but also of their activation through quantification of HER homo- or heterodimers. First, EGFR and HER2 expression levels were quantified in 18 breast tumors and the results were compared with those obtained by using reference methods. The EGFR number per cell determined by TR-FRET was significantly correlated with *EGFR* mRNA copy number (*P*<0.0001). Moreover, our method detected HER2 overexpression with 100% specificity and sensibility, as confirmed by the standard IHC, FISH and qPCR analyses. EGFR and HER2 dimerization was then assessed, using as controls xenograft tumors from cell lines with known dimer expression profiles. Our results show that quantification of HER dimerization provides information about receptor activation that cannot be obtained by quantification of single receptors. Quantifying HER expression and dimerization by TR-FRET assays might help identifying novel clinical markers for optimizing patients’ treatment in oncology.

## Introduction

The human epidermal growth factor receptor 2 (HER2) is a 185 KDa transmembrane glycoprotein that belongs to the human epidermal receptor (HER) family of receptor tyrosine kinases [1]. In 1987, Slamon and colleagues first reported that amplification of the *HER2* gene occurs in about a third of human breast cancers and correlates with poor prognosis [2]. Subsequent *in vitro* and *in vivo* studies characterized the biological consequences of this molecular abnormality, demonstrating that *HER2* is a potent oncogene that promotes tumor growth, angiogenesis and metastasis [3]–[5]. It was thus hypothesized that HER2 inhibition could be an effective therapeutic strategy for the treatment of HER2 overexpressing tumors. This led to the development of trastuzumab, a specific anti-HER2 humanized recombinant monoclonal antibody (mAb), which showed considerable clinical utility in patients with HER2-overexpressing breast tumors in both metastatic [6]–[10] and adjuvant [11]–[13] settings.

Concomitantly, theranostic tests were developed to assess HER2 status in order to identify patients who might benefit from trastuzumab. The United States Food and Drug Administration approved immunohistochemistry (IHC) staining for detecting HER2 protein overexpression and fluorescence in situ hybridization (FISH) assays for quantifying *HER2* amplification [14]. However, they are not sufficient for optimal patients’ selection, as less than half of the patients with HER2-positive cancers will respond to trastuzumab therapy [9], [10], [15]. It is believed that HER2 overexpression causes aberrant activation of intracellular signaling pathways through spontaneous formation of HER2 homodimers and/or increased heterodimerization with other members of the HER family, such as the epidermal growth factor receptor (EGFR). As these receptors can display distinct signaling properties dependent on their dimerization partner [16], quantification of HER dimers could help predicting the patient’s response and outcome to anti-HER therapies. However, only few works, if any, have investigated the use of HER dimer expression profile to stratify patients into subgroups who might respond differently to trastuzumab.

Conventional approaches to detect HER dimers rely on immunoprecipitation and chemical crosslinking techniques [17] that have low throughput and are limited to *in vitro* studies, as they require large amount of proteins. To overcome these limitations, we have developed TR-FRET assays for quantifying HER dimerization in patients’ samples. The TR-FRET technology combines Förster resonance energy transfer (FRET) with time-resolved (TR) detection. FRET relies on the transfer of energy between two suitable fluorophores, a donor and an acceptor. Excitation of the donor by an energy source triggers energy transfer to the acceptor only if they are in close proximity and the acceptor will then emit fluorescence. The use of long-lived fluorophores combined with TR detection that introduces a delay between the excitation pulse and the emission detection allows the suppression of short-lived background fluorescence and improves the sensitivity of FRET-based assays [18]. To quantify HER dimers, we used anti-HER antibodies coupled with either donor or acceptor fluorophores. When two labeled antibodies that form a FRET pair bind to two receptors that form a dimer, the distance between the donor and the acceptor is small enough to allow FRET to occur. The intensity of the acceptor fluorescence signal measured in TR mode is proportional to the number of dimers, thereby allowing quantitative measurements. We also developed TR-FRET assays for quantifying the expression of individual HER, by using antibody pairs that recognize two distinct epitopes in a single receptor. In this study, we examined the reliability of these new TR-FRET assays for the analysis of tumor cryosections. For this purpose, we first quantified EGFR and HER2 expression in eighteen breast tumors and confirmed our results using established techniques. Then, we assessed EGFR:EGFR, HER2:HER2 and EGFR:HER2 dimer levels, using as controls xenograft tumors from cell lines with known dimer expression profiles. Such analysis provided information about EGFR and HER2 activation that cannot be obtained by quantification of single receptors.

## Results

### Design and Optimization of the TR-FRET Assays

**Figure 1:**
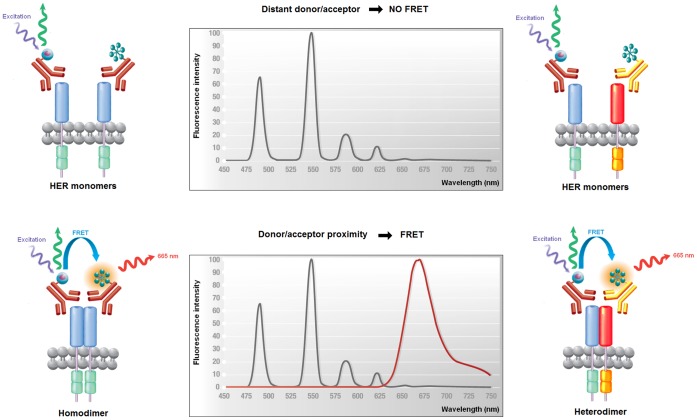
Quantification of HER dimers using the TR-FRET technology. When an antibody conjugated with Lumi4® Tb (donor fluorophore) and an antibody coupled to d2 (acceptor fluorophore) are brought together by a biological interaction, a portion of the energy captured by Lumi4® Tb during excitation is released through fluorescence emission, while the remaining energy is transferred to d2, which re-emits a specific long-lived fluorescence at 665 nm. In the case of homodimer quantification, the fluorescence emitted will represent only 50% of the actual amount of homodimers due to the formation also of homodimers that interact with two antibodies that are both labeled with the donor or the acceptor fluorophore and that are, therefore, undetectable.

**Figure 2:**
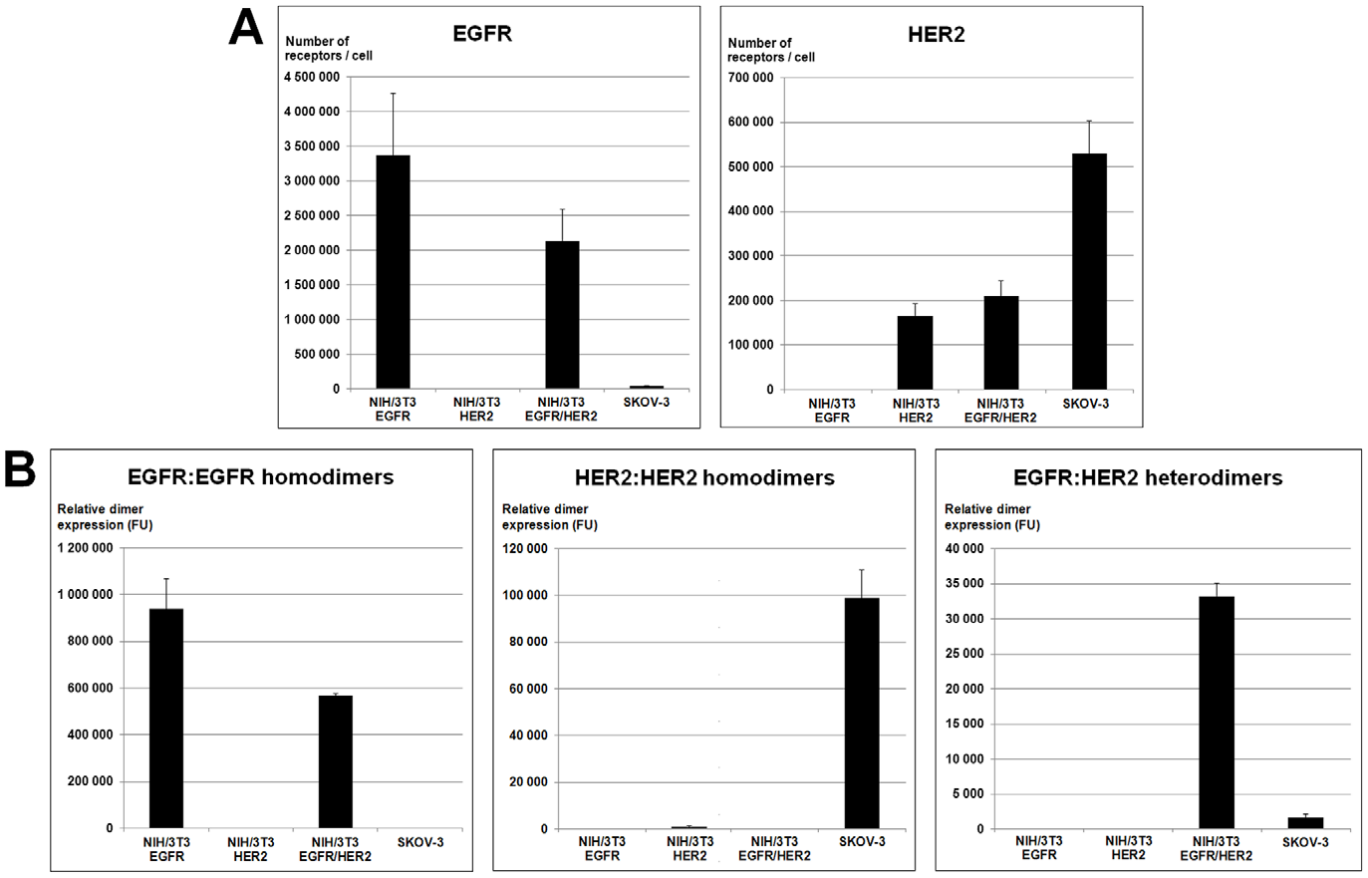
Characterization of the tumor xenograft models using the developed antibody-based TR-FRET assays. (a) Absolute quantification of EGFR and HER2 expression in NIH/3T3 EGFR (high EGFR expression, no HER2 expression), NIH/3T3 HER2 (high HER2 expression, no EGFR expression), NIH/3T3 EGFR/HER2 (high EGFR and HER2 expression) and SKOV-3 (low EGFR expression, high HER2 expression) tumor xenografts using the HER quantification assays. (b) Detection of EGFR:EGFR, HER2:HER2 and EGFR:HER2 dimers using the HER dimer quantification assays.

We developed TR-FRET assays to accurately quantify HER homo- and hetero-dimers (Fig. 1 for a schematic description of the TR-FRET principle). We also designed assays for quantifying EGFR and HER2 absolute expression by using mAb pairs directed against two distinct epitopes of each receptor. The key steps of each assay (antibody selection and labeling, reaction conditions and reagent concentrations) were optimized in preliminary experiments using tumor xenografts of cells expressing HER2 or/and EGFR (data not shown). The concentration of labeled antibodies was the key parameter for assay sensitivity. Specifically, we tested antibody concentrations from 5 to 150 nM and found that with 50 nM of each antibody the specific TR-FRET signal reached a plateau value and was approximately 20-fold higher than when using 5 nM of each antibody. In contrast to traditional TR-FRET assays in which sub-nanomolar to nanomolar concentrations of labeled antibodies are used, higher concentrations of Lumi4® Tb-labeled antibodies require the addition of a washing step to avoid saturation of the detector and to minimize the donor long-lived background in the acceptor channel (665 nm). Furthermore, to normalize the TR-FRET signal to the amount of biological material under study, sections were stained with the DNA-binding fluorescent dye Hoechst 33342, which also requires a washing step to reduce the background due to unbound stain. After the last wash, tissues were sonicated in order to obtain a homogeneous lysate which could be transferred into microplate wells. All data presented hereafter were obtained with this optimized protocol that is described in detail in the Materials and Methods section. To quantify the absolute amount of EGFR and HER2 using the HER quantification TR-FRET assays, we used the tumor xenograft models as calibrators to convert the fluorescence signal measured by TR-FRET analysis into the number of receptors per cell (Fig. 2A). To this aim, first, we tried to estimate by fluorescence-activated cell sorting (FACS) the number of receptors per cell in cell suspensions obtained by dissociation of NIH/3T3 EGFR, NIH/3T3 HER2, NIH/3T3 EGFR/HER2 and SKOV-3 tumor xenografts. However, this was not possible because the HER extracellular domain was degraded following the enzymatic treatment required for extracellular matrix disruption (data not shown). Therefore, we quantified the number of EGFR and HER2/cell in the cultured cell lines and assumed that the tumor xenografts derived from these cells had similar HER expression levels.

In addition, these tumor xenograft models were also used as positive and negative controls for the dimer quantification assays because the HER dimer expression profiles of the xenografted cell lines were already known. Indeed, in a previous work [19], we showed that EGFR-overexpressing cells (NIH/3T3 EGFR and NIH/3T3 EGFR/HER2 cells) have high EGFR:EGFR homodimer levels. Conversely, HER2-overexpressing cells (SKOV-3, NIH/3T3 HER2 and NIH/3T3 EGFR/HER2 cells) do not systematically display high levels of HER2:HER2 homodimers. Specifically, SKOV-3 cells have high HER2:HER2 expression, while they are barely detectable in NIH/3T3 HER2 and NIH/3T3 EGFR/HER2 cells. Cells that overexpress both EGFR and HER2 (NIH/3T3 EGFR/HER2 cells) are characterized by high levels of EGFR:HER2 heterodimers, whereas cells that display high levels of HER2 and low levels of EGFR (SKOV-3 cells) have low EGFR:HER2 expression. Dimer quantification by TR-FRET in tumor xenografts (Fig. 2B) gave results that were in agreement with those obtained in the corresponding cultured cell lines, thus confirming the suitability of our assays for the analysis of tumor samples.

### Absolute Quantification of EGFR and HER2 Protein Expression by TR-FRET in Patients’ Samples

To assess the suitability of our TR-FRET assays for HER quantification in patients’ samples, we analyzed EGFR and HER2 expression in 18 breast tumors (Fig. S1). The median absolute expression levels were 2 800 (range 220–35 500) EGFR/cell and 49 800 (range 11 500–584 000) HER2/cell. Five (27.8%) of the 18 tumors expressed very high levels of HER2 (216 800, 234 300, 391 000, 491 500 and 584 000 HER2/cell). On average, HER2 expression levels were 66-fold higher than those of EGFR. We tested the reproducibility of the assay results by replicating the experiments three times for each sample. The average coefficients of variation (CV) were 22% for the EGFR and 19% for the HER2 quantification assay. This variability takes into account the biological variability because different cryosections were used for each experiment.

**Figure 3:**
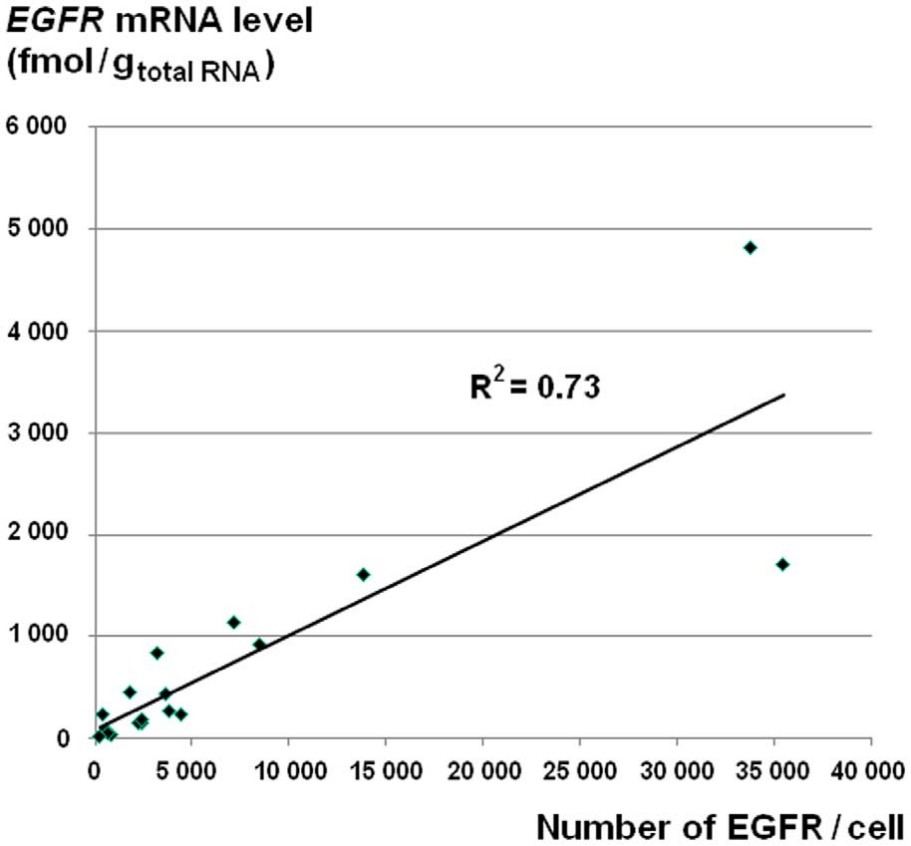
Correlation between TR-FRET and RT-qPCR measurements of EGFR expression. EGFR protein expression determined by TR-FRET was plotted against *EGFR* mRNA copy number measured by RT-qPCR. With a determination coefficient (R^2^) of 0.73, the resulting curve demonstrates a linear correlation between the measurements of the two assays.

To confirm the EGFR quantification, we extracted total RNA from each breast tumor for RT-qPCR analysis. We found a positive linear correlation (Rho = 0.84, *P*<0.001) between the EGFR protein expression levels determined by TR-FRET and the *EGFR* mRNA expression levels measured by RT-qPCR (Fig. 3).

**Figure 4:**
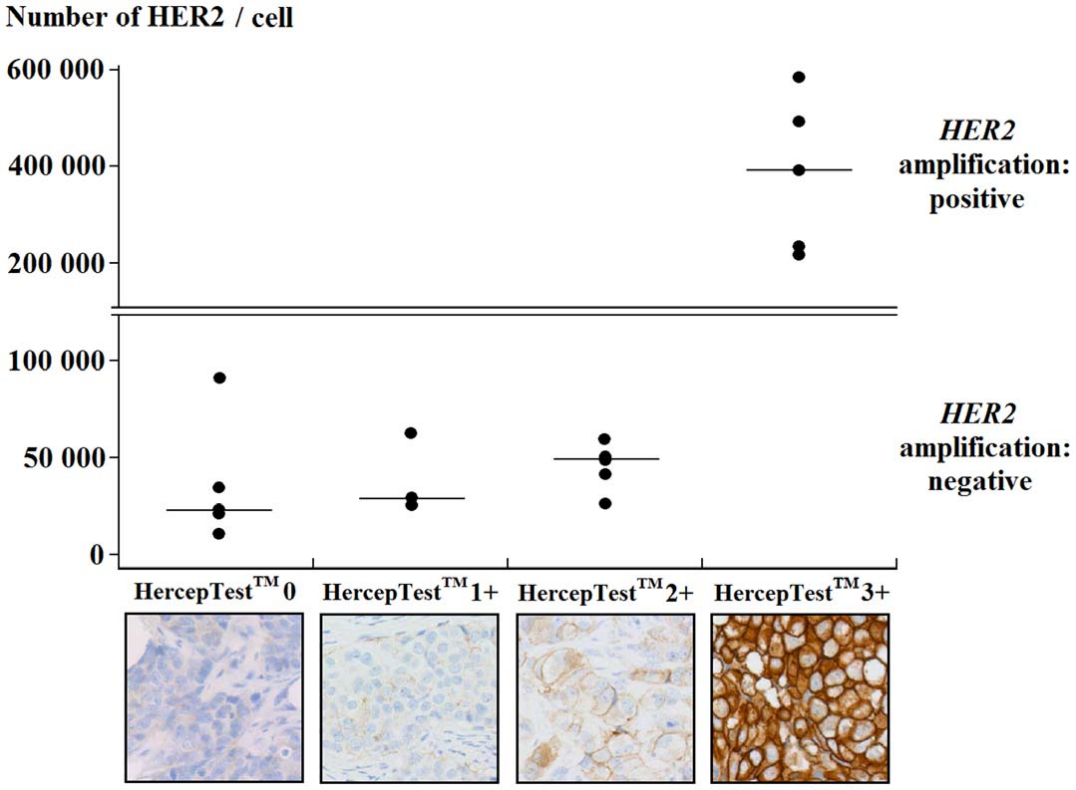
Comparison of our TR-FRET assay with standard techniques for the assessment of HER2 status. The number of HER2/cell was evaluated by TR-FRET in breast tumors stratified according to their Herceptest™ score and the *HER2* gene amplification status. *HER2* amplification was determined by FISH and confirmed by qPCR. The Herceptest™ scoring system was as follows: 0, no staining or membrane staining in <10% of tumor cells; 1+, faint/barely perceptible membrane staining in >10% of tumor cells; 2+: weak to moderate complete membrane staining in >10% of tumor cells, or strong complete membrane staining in 10–30% of tumor cells; 3+, strong complete membrane staining in >30% of tumor cells.

To validate the results of the HER2 quantification assay, we evaluated HER2 expression using the HercepTest™ and *HER2* amplification by FISH and qPCR analyses (Fig. 4). There was no overlap in the TR-FRET expression levels of HER2 between HER2-positive and HER2-negative tumors. Only the five breast tumors with >150 000 HER2/cell had a HercepTest™ score of 3+ and were positive for *HER2* gene amplification. This indicates that our TR-FRET method can detect HER2 overexpression with 100% specificity and sensitivity.

Taken together, these results validate our TR-FRET assays for the absolute quantification of EGFR and HER2 expression in tumor samples.

### TR-FRET Quantification of HER Dimers in Patients’ Samples

EGFR:EGFR homodimers were detected in 5 (27.8%) of the 18 breast tumors, HER2:HER2 homodimers in 12 (66.7%) and EGFR:HER2 heterodimers in 4 tumors (22.2%) (Fig. S2). In most of these tumors, dimer expression levels were low to medium (range 200–2700 FU), except for five tumors that showed high levels of HER2:HER2 homodimers (range 12 000–32 400 FU). For each sample, we calculated the CV of the EGFR:EGFR, HER2:HER2 or EGFR:HER2 quantification in three independent experiments and obtained a mean value <25% for the three assays.

**Figure 5:**
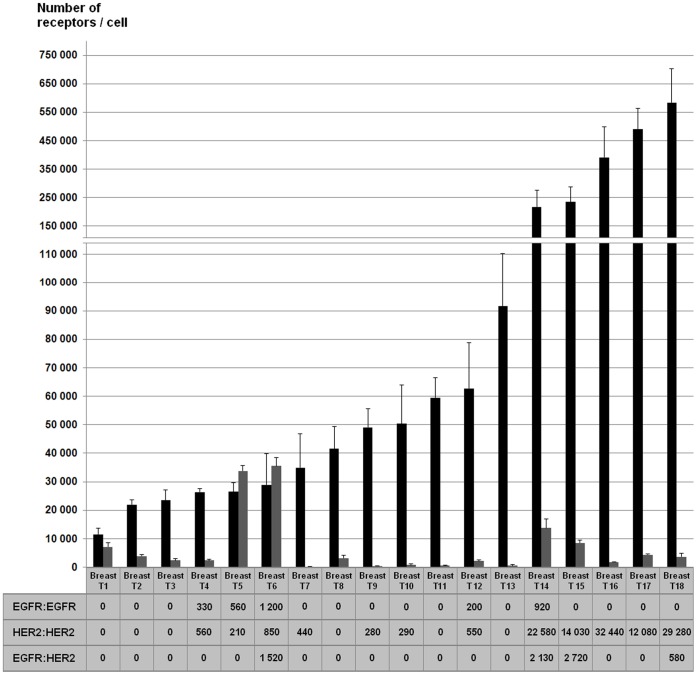
Comprehensive TR-FRET analysis of EGFR and HER2 expression and dimerization in 18 breast tumors. The histogram describes the expression of HER2 (black bars) and EGFR (grey bars) in 18 breast tumors. The table indicates the expression levels of EGFR:EGFR, HER2:HER2 and EGFR:HER2 dimers, which are expressed in FU.

The Spearman’s test showed an overall weak correlation between EGFR and EGFR:EGFR (Rho = 0.53, *P* = 0.022), EGFR and EGFR:HER2 (Rho = 0.58, *P* = 0.012) as well as between HER2 and HER2:HER2 expression levels (Rho = 0.66, *P* = 0.003). Nevertheless, samples with comparable EGFR and HER2 levels (Breast T5 and Breast T6) showed distinct dimer patterns (Fig. 5). Moreover, although all HER2-overexpressing tumors had high levels of HER2:HER2 homodimers, our results indicate that these tumors could be separated into subgroups according to the presence of EGFR:HER2 heterodimers and/or EGFR:EGFR homodimers.

## Discussion

We developed TR-FRET-based assays for the accurate quantification of EGFR and HER2 expression in tumor cryosections, as well as assays for the assessment of their activation though the quantification of receptor homo- or hetero-dimerization. As our protocol includes washing steps, it differs from the classical TR-FRET based assays, such as HTRF® (homogeneous time-resolved fluorescence). In HRTF®, the concentration of labeled antibodies is limited to approximately 5 nM. At higher concentrations, dynamic FRET would occur, thus impairing the specificity of the signal. Removal of unbound antibodies by washing steps allowed us to raise the antibody concentrations to 50 nM. This increased the specific signal by about 20-fold and thus significantly improved the sensitivity of the assays. The use of a non-homogeneous format also offers the advantage of allowing the addition of a DNA staining step with a fluorescent dye to quantify the amount of biological material. Indeed, differently from the cell-based TR-FRET assays developed for drug discovery applications, in which a known number of cells are initially loaded in the wells of microplates, in our assays the actual amount of biological material is unknown (tumor cryosections). Therefore, the sample cell number has to be evaluated simultaneously with the TR-FRET analysis in order to normalize the measured receptor or dimer levels to the amount of biological material. For this purpose we selected the Hoechst dye which gives a fluorescent signal proportional to the amount of DNA and consequently to the number of cells. Such normalization has been described for the quantification of G protein-coupled receptor kinases expression in transfected cells [20] and for the correction of well-to-well cell density variations in drug screening experiments [21].

The NIH/3T3 EGFR, NIH/3T3 HER2, NIH/3T3 EGFR/HER2 and SKOV-3 tumor xenograft models were used as calibrators for the HER quantification assays in order to convert the TR-FRET signal into the number of receptors per cell. As we could not estimate directly the number of HER per cell in the xenografts, we quantified the number of EGFR and HER2/cell in the relevant cultured cell lines and assumed that the xenografts derived from these cells had similar HER expression levels. Considering that the cell lines used for calibration overexpress EGFR and/or HER2, the number of these receptors per cell is unlikely to differ significantly between cultured cells and tumor xenografts.

After calibration of the HER quantification TR-FRET assays using these tumor xenograft models, we analyzed 18 breast tumors. To our knowledge, this is the first time that the number of HER per cell is estimated in patients’ samples. Such absolute quantification allows comparing the expression levels of EGFR and HER2 in the same tumor. The reliability of our results was confirmed using several established techniques, such as RT-qPCR, qPCR, FISH and IHC.

In the routine clinical practice, HER2 quantification is required for selecting patients with breast cancer who are eligible for trastuzumab therapy because this drug is effective only in HER2-overexpressing tumors [8], [10], [22], [11]. Usually, HER2 testing is performed by IHC (protein expression) and FISH (gene amplification). IHC is a semi-quantitative methodology that scores HER2 expression on a scale from IHC 0 (negative) to IHC 3+ (strongly positive), based on the percentage of stained malignant cells and the degree of membrane staining in these cells. However, IHC reliability is questionable, since discordant results may be obtained in different laboratories [23], possibly due to the subjective aspect of the IHC scoring system (observer bias). Due to its more objective scoring system, FISH is considered to be more reliable than IHC, but it requires more pathologist’s interpretation time and is more expensive. Besides, FISH provides only an indirect measure of HER2 protein expression, which is the trastuzumab target. HER2 quantification by TR-FRET incorporates the advantages of both IHC and FISH, while eliminating many of their drawbacks. Indeed, it is a quantitative method for determining HER2 protein levels, which are automatically measured by a computer. Moreover, our approach allows the distinction of subtle differences in HER2 expression levels that are distributed over a larger dynamic range than the four classes of the IHC scoring system.

In addition, besides the absolute quantification of EGFR and HER2 expression, our TR-FRET assays also allow assessing the activation of these receptors through the quantification of homo- and hetero-dimers. We previously validated the use of TR-FRET for the analysis of HER dimers in stably transfected NIH/3T3 cell lines that express EGFR and/or HER2 and in various tumor cell lines [19]. Here, we developed a protocol adapted to the analysis of tumor cryosections. Our results show that breast tumors with elevated levels of EGFR or HER2 are more likely to express dimers. However, distinct dimer patterns were observed in samples with similar EGFR and HER2 levels. In addition, tumor xenografts from the NIH/3T3 HER2 and NIH/3T3 EGFR/HER2 cell lines had high levels of HER2, whereas HER2 homodimers were almost undetectable. Taken together, these results indicate that quantification of EGFR and HER2 protein expression is not sufficient to predict if and what type of dimers are formed. Hence, quantification of HER dimers provides additional and specific information that cannot be obtained through the quantification of the single receptors.

As a predictive marker, HER2 status is not sufficient for optimal patient selection for trastuzumab therapy. Indeed, only 30% of patients with HER2-positive tumors benefit from trastuzumab in the metastatic setting and only 50% of patients in the adjuvant setting [24]. Our results show that subgroups of HER2 overexpressing tumors can be identified based on the presence of EGFR:HER2 heterodimers or EGFR:EGFR homodimers. These tumors may respond differently to trastuzumab treatment. To verify this hypothesis, we are currently performing a retrospective study to assess the predictive role of HER dimer quantification using our TR-FRET assays in patients with breast cancer treated with trastuzumab-based therapies. Moreover, if dimerization reflects receptor activation, then quantification of HER2 homodimers may help to better identify patients that are eligible to anti-HER2 therapy. In our series of 18 breast tumors, HER2:HER2 homodimers were detected in two-third of the cases. Specifically, they were observed not only in the tumors that overexpress HER2 (>150 000 receptors/cell), but also in about half of the tumors with low HER2 expression (HercepTest™ 0, 1+ or 2+), albeit at levels that were on average 50-fold lower than in the HER2 overexpressing tumors.

As conventional approaches to analyze protein-protein interactions, such as co-immunoprecipitation or cross-linking, are not suitable for clinical material, novel methods have been proposed. Monogram Biosciences has developed the VeraTag™ assay [25], [26] to quantify HER proteins and dimers in FFPE tissue samples through the release of fluorescent tags conjugated to specific anti-HER antibodies that requires proximity to a second HER antibody. However, this technology is cumbersome and unavailable to research or clinical laboratories and samples must be shipped to Monogram for the analysis. The Duolink™ *in situ* proximity ligation assay [27] detects HER dimers by using antibodies coupled to oligonucleotides. When two target proteins are separated by less than 40 nm, a complex succession of hybridization/amplification steps leads to the visualization of HER dimers using *in situ* fluorescence microscopy. In our TR-FRET assays, a signal is obtained when the distance between the acceptor and donor fluorophores is less than 12 nm. This length difference is significant, because by using a technology that detects proximity of 40 nm, it becomes difficult to distinguish a dimer from two co-localized receptors that do not interact. Our results (absence or very low HER2:HER2 expression in the NIH/3T3 HER2 and NIH/3T3 EGFR/HER2 cell lines) indicate that our dimer quantification assays can make this distinction.

In conclusion, our study demonstrates the feasibility of antibody-based TR-FRET assays as an efficient and comprehensive approach to study not only the absolute expression of membrane receptors, but also their functional dimerization in patients’ tumor samples. This technology may offer the opportunity to better predict patients’ response to HER-targeted agents, thus allowing tailored therapy.

## Materials and Methods

### Patients’ Samples and Ethics Statement

Eighteen breast tumor samples were obtained from the tissue biorepository of Asterand (Royston, United Kingdom). Asterand ensures that all relationships through which Asterand acquires donated human tissues and through which it supplies such tissues to third parties are governed by legal agreements, and are subject to stringent ethical review by Asterand and/or by appropriate federal, state and local institutions or review boards. The informed consent for the use of clinical records and biological samples for research purposes was obtained from all patients.

### Tumor Xenografts

The ovarian carcinoma SKOV-3 cell line, in which *HER2* is amplified, and the transgenic cell lines NIH/3T3 EGFR, NIH/3T3 HER2 and NIH/3T3 EGFR/HER2, which are mouse embryonic fibroblasts stably transfected with *EGFR* and/or *HER2* [19] were xenografted in nude athymic mice. The xenograft tumors were used as control for optimization and validation of the TR-FRET assays as the dimer expression profiles of these cell lines are known [19].

### TR-FRET Assays

Lumi4® Tb and the d2 dye (Cisbio Bioassays, Codolet, France) were used as donor and acceptor fluorophores, respectively. Lumi4® Tb is a trademark of Lumiphore Inc. For EGFR quantification, the TR-FRET assay was performed using Lumi4® Tb-labeled cetuximab and d2-labeled Ab-10, two mAbs that recognize distinct EGFR epitopes. Similarly, HER2 expression was quantified using Lumi4® Tb-labeled trastuzumab and d2-labeled FRP5. TR-FRET assays for the detection of EGFR:EGFR and HER2:HER2 homodimers were designed using a single antibody (cetuximab or trastuzumab) labeled with Lumi4® Tb or d2 to form a FRET pair. EGFR:HER2 heterodimers were quantified using the anti-EGFR 528 mAb labeled with Lumi4® Tb and the d2-labeled FRP5 (anti-HER2 mAb). Cetuximab, Ab-10 and 528 were purchased from Merck KGaA (Darmstadt, Germany), Thermo Scientific (Cergy Pontoise, France) and the American Type Culture Collection (ATCC), respectively. Trastuzumab was from Roche Pharma AG (Grenzach-Wyhlen, Germany). FRP5 [28] was kindly provided by Nancy Hynes (Basel, Switzerland).

For all TR-FRET assays, 50 µm thick tumor cryosections were incubated in 180 µl TR-FRET buffer (1× PBS/10% BSA) containing 50 nM of each antibody overnight. Then, DNA was stained by adding 20 µl of 0.1 mg/mL Hoechst 33342 (Invitrogen, Cergy Pontoise, France) at room temperature for 10 min. After washing and centrifugation, samples were resuspended in TR-FRET buffer, sonicated and transferred into a microplate. The fluorescence signals of Lumi4® Tb and d2 were measured respectively at 620 and 665 nm (in TR mode: delay, 60 µsec; time gate, 400 µsec) upon 337 nm excitation using a Pherastar™ FS fluorometer (BMG LABTECH, Champigny-sur-Marne, France). The Hoechst 33342 signal was measured in fluorescence mode at 460 nm. The fluorescence emissions (F) from the samples at 665 nm, 620 nm and 460 nm were corrected for background fluorescence using the formula: F_corrected_ = F_sample_ - F_background_, where the F_background_ values were obtained by reading the fluorescence of the TR-FRET buffer.

Moreover, for each assay, the fluorescence signals of serial dilutions of a solution containing 50 nM of Lumi4® Tb- and d2-labeled antibodies were measured simultaneously with the samples and the 665 nm emission was plotted against the 620 nm emission. The resulting curve was used to compute the fluorescence contribution from Lumi4® Tb at 665 nm (F665_Tb_) using the 620 nm emission of the samples. The TR-FRET signal was expressed as ΔF665 =  F665_sample_ - F665_Tb_. With an average value of 100 000 fluorescence units (FU), the Hoechst 33342 fluorescence emission at 460 nm (F460) was used to normalize the TR-FRET signal relatively to the cell content: TR-FRET_normalized = _ΔF665/(F460/100 000). The normalized TR-FRET signal was expressed in FU.

In order to convert the normalized TR-FRET signal into the number of receptors per cell for the absolute quantification of EGFR and HER2 in tumor samples, the number of these receptors per cell was first quantified in the NIH/3T3 EGFR, NIH/3T3 HER2, NIH/3T3 EGFR/HER2 and SKOV-3 cell lines. For that purpose, cultured cells were analyzed by FACS using a quantitative immunofluorescence indirect assay (QIFI kit, Dako, Cambridge, United Kingdom) as previously described [19]. Then, EGFR and HER2 expression were measured in the corresponding tumor xenografts using the TR-FRET assays. Thus, based on the assumption that EGFR and HER2 are expressed at comparable levels in the cultured cell line models and in the tumor xenografts derived from these cells, the xenograft models could be used as calibrators to convert the TR-FRET signal into the number of receptors per cell.

### Quantification of *EGFR* mRNA Levels and *HER2* Copy Number

RNA and DNA were isolated concomitantly from breast tumors using the RNeasy Mini Kit (Qiagen, Courtaboeuf, France) and the QIAamp DNA Mini Kit (Qiagen) according to a modified protocol [29]. The absolute mRNA expression levels of *EGFR* were quantified by reverse transcription-quantitative polymerase chain reaction (RT-qPCR) as previously described [29]. The average number of *HER2* gene copies was evaluated by qPCR, using *β-Actin* as reference gene. Normal human genomic DNA and DNA isolated from the SKBR-3 breast cancer cell line (from ATCC) were used as controls for *HER2* non-amplification and *HER2* amplification, respectively. Primer sequences are available upon request. The RT-qPCR and qPCR assays were performed with the Rotor-Gene Q (Qiagen) using the QuantiFast™ SYBR® Green PCR Mix (Qiagen). Amplification started with an enzyme activation step at 95°C for 5 minutes, followed by 40 cycles consisting of 10 seconds of denaturation at 95°C and 30 seconds of annealing/extension at 60°C.

### HER2 Expression by IHC and FISH Analysis of *HER2* Copy Number

IHC and FISH analysis of HER2 in the 18 breast tumors were performed by an independent clinical laboratory (Asterand) using the HercepTest™ (Dako) and the *HER2* FISH pharmDx™ Kit (Dako), respectively. The HercepTest™ was carried out on formalin-fixed paraffin-embedded (FFPE) samples that are the mirror image of the frozen breast tumor samples used for the TR-FRET assays, following the new guidelines recently published by the American Society of Clinical Oncology [30]. Accordingly, only samples with more than 30% of tumor cells (rather than the originally specified 10%) with strong immunostaining for HER2 were classified as 3+.

### Statistical Analysis

The relationship between EGFR expression levels measured by RT-qPCR or TR-FRET and the correlations between receptor and dimer expression levels were assessed using a linear regression model and the Spearman’s test. Differences were considered statistically significant when *P*<0.05. Statistical analysis was performed using the STATA 11.0 software (StataCorp LP, College Station, TX, USA).

## Supporting Information

Figure S1 **TR-FRET quantification of EGFR and HER2 protein expression in 18 breast tumors.**
(TIF)

Figure S2 **TR-FRET quantification of HER dimers in 18 breast cancers.**
(TIF)
