# Supplementary figures and images for: Quantification of HER Expression and Dimerization in Patients’ Tumor Samples Using Time-Resolved Förster Resonance Energy Transfer
Source: PLoS One. 2012 Jul 19;7(7):e37065. doi: 10.1371/journal.pone.0037065 (PMC3400639; doi:10.1371/journal.pone.0037065)

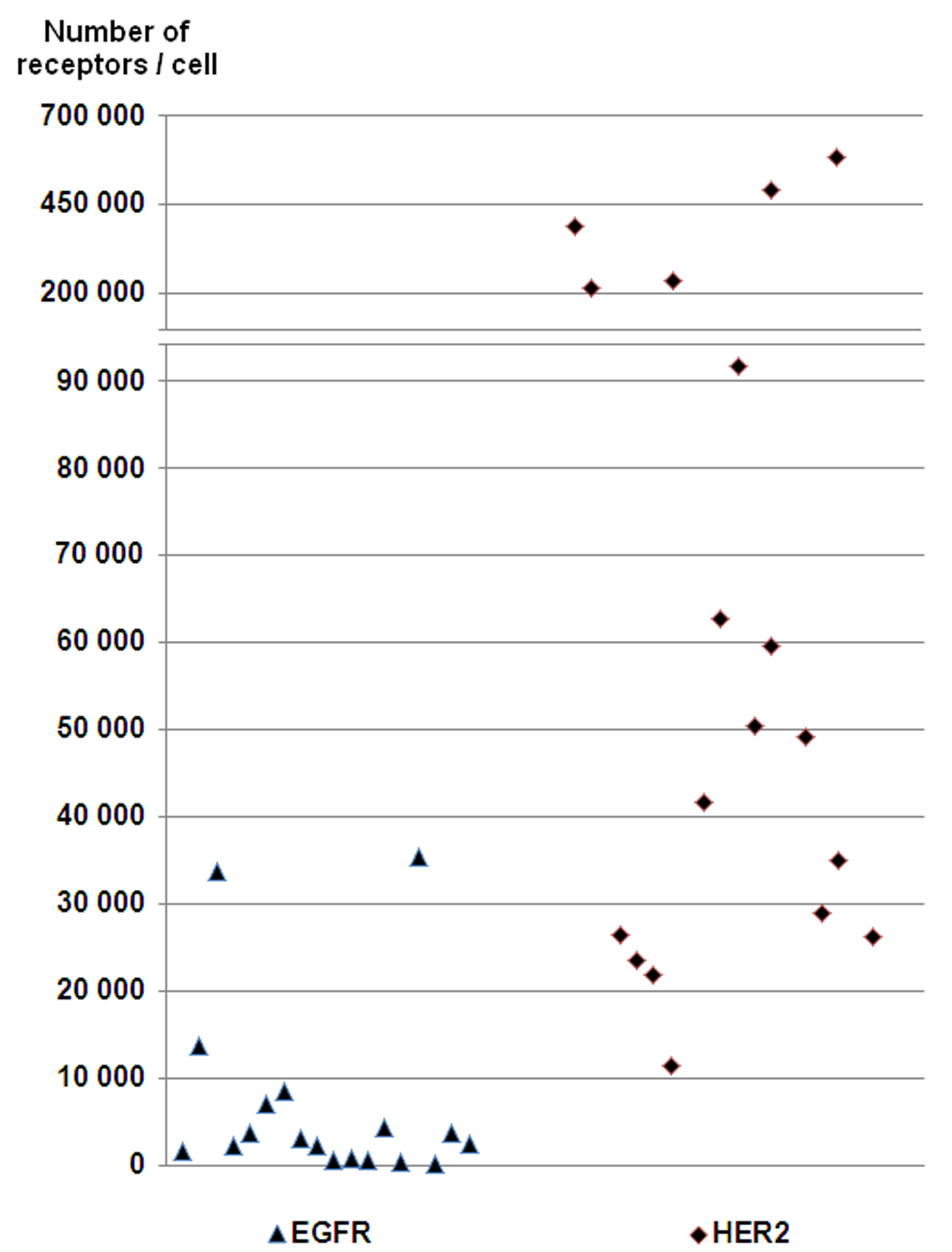

Supplement: Figure S1 — TR-FRET quantification of EGFR and HER2 protein expression in 18 breast tumors. (TIF) [file pone.0037065.s001.tif]

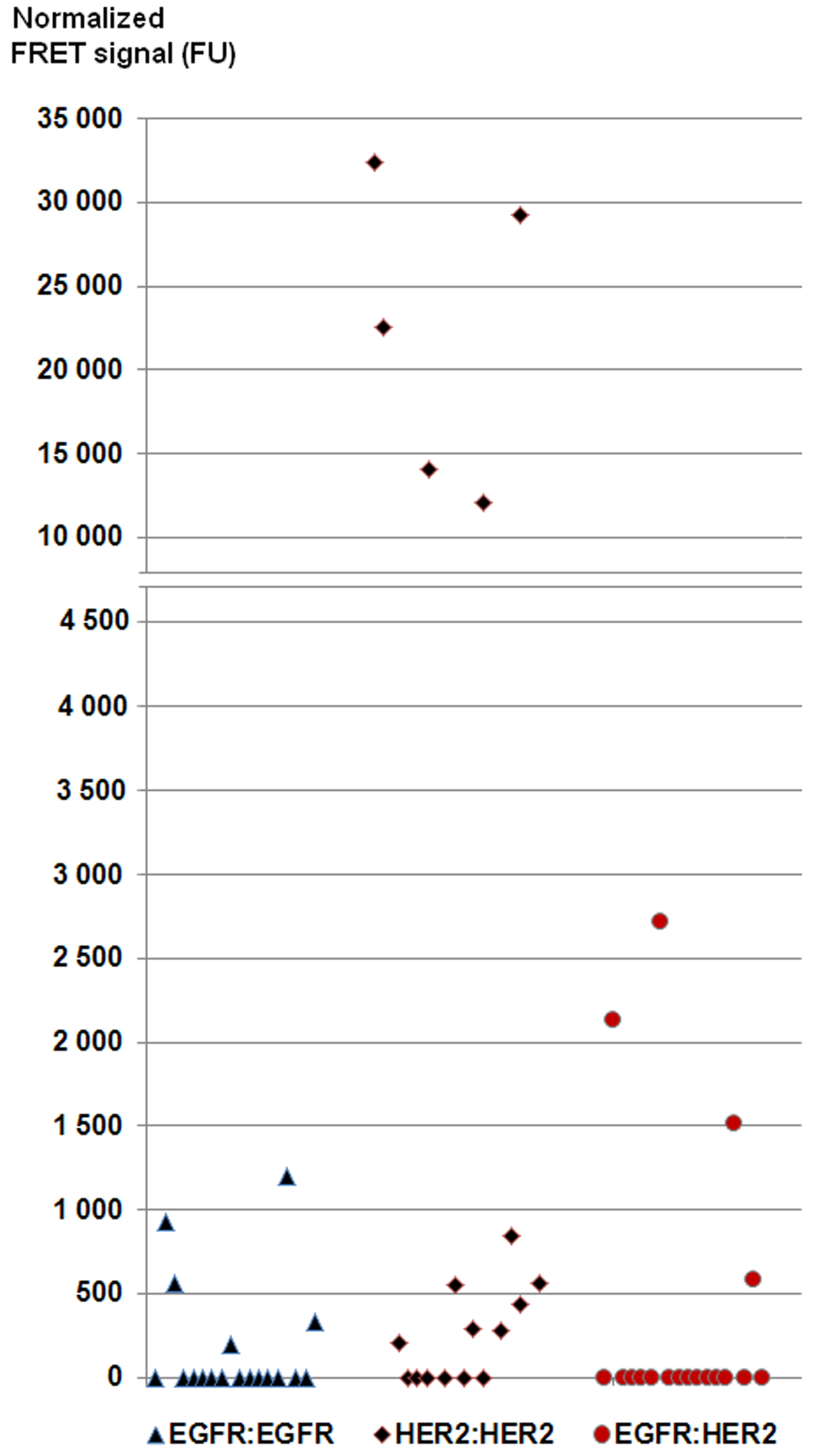

Supplement: Figure S2 — TR-FRET quantification of HER dimers in 18 breast cancers. (TIF) [file pone.0037065.s002.tif]
